# Buffalo Telephonic Exchange

**Published:** 1879-04

**Authors:** 


THE BUFFALO TELEPHONIC EXCHANGE is now in active operation, thus connecting us with all parts of the city, and capable of doing admirable service. It is greatly to be regretted that there should be competing lines to the disadvantage of both Companies, and diminished value to subscribers. We hope this evil will soon be abated. Telephonic communication is the wonderful discovery of the age.

